# Exploring home-based care nurses’ mindset for nursing practices: a phenomenological study

**DOI:** 10.1186/s12912-022-01068-w

**Published:** 2022-11-01

**Authors:** Bodil Aarmo Brenne, Marianne Hedlund, Kari Ingstad

**Affiliations:** 1 Faculty of Nursing and Health Science, Nord university, Pb. 93, 7601 Levanger, Norway; 2 Department of Social Work, NTNU, 7491 Trondheim, Norway

**Keywords:** Care Trajectory Management, Clinical mindlines, Home Care, Home nursing, Nurse role, Professional Independence, Situational awareness

## Abstract

**Background:**

Home nursing is an essential aspect of healthcare and can address future health challenges. The nature of nursing and its practical applications are of particular interest, as nursing involves technical knowledge, rational procedures, and diverse skills. It is consequential to explore nursing practices in context to understand how nurses navigate their work. This study aimed to explore the characteristics of home care nurse practices and how nurses solve tasks in the context of nursing in home care.

**Methods:**

This is an exploratory qualitative research study using a descriptive phenomenological approach. We reported following the COREQ guidelines. Fifteen in-depth interviews with nurses from four Norwegian municipalities were conducted and analysed according to stepwise inductive analysis.

**Results:**

The analysis revealed three main patterns that characterise nursing practices in home care: ‘To be vigilant’, ‘To be an all-rounder’, and ‘To act with independence’. The content and distinction of these patterns are discussed through a theoretical framework of ‘clinical mindlines’. There are multiple mindlines and complex realities for home-based care nursing. The nurses displayed great sensitivity in their practice, were knowledgeable about where they focused their attention, adapted their actions to the context, and demonstrated their independence as professionals.

**Conclusion:**

Nurses’ vigilance and contextual insight are critical to their practice approach and task-solving abilities. These professionals need to manage emergent organisations and exercise independence and professional judgment when adapting their work to the context of home care patients. Future health policy should not strictly be based on standardised guidelines; depending on the context, it is also appropriate to focus on nurses’ practical knowledge and the importance of mindlines.

## Background

Home nursing is an essential aspect of healthcare that can meet future health challenges, and new knowledge is necessary to address these challenges sustainably [1]. Home nursing refers to the provision of healthcare in private homes rather than at a hospital or clinic. There is a worldwide focus on the reorganisation of health and social care services in this context [2, 3] due to the growing needs of an ageing population and increasing demand for long-term care [4, 5].

The development and provision of home-based health and social care must achieve a balance between the limited resources and increased expectations for the high-quality, individualised care mandated today [6]. Shorter hospital stays seem to be a global trend [7, 8], including in Norway, where healthcare reforms have increasingly pressured primary health services to implement more complex and comprehensive treatment for patients previously being treated by specialist health services [9].

Norwegian health services posit that healthcare should be available to all. Norway adheres to a comprehensive welfare model, with home care having been available for many decades [10]. The state draws up national health policies and allocates resources while local authorities are responsible for the planning and provision of healthcare [11, 12]. The implementation of a new public management system into the healthcare system focuses on cost reduction and the formation of different result units. This system is expected to address financial challenges in the healthcare system, thereby working towards the goal of equitable healthcare distribution.

The most recent major health reform in Norway, called the Coordination Reform, aims to improve the collaboration between different healthcare stakeholders and levels in terms of prevention, early intervention, early diagnosis, treatment, and follow-up care [13]. While coordinating health services for this purpose is a challenge in many countries, home nursing would play an important role in its realisation.

There may be variations in what home nursing entails across countries and regions due to differences in organisations and structure. In Norway, municipalities are responsible for providing home nursing to those who need it, regardless of age or diagnosis. Home nursing includes assistance with medicines or medication management, nursing care such as wound care, and observations by a healthcare professional. Home nursing is free of charge in Norway and is provided by registered and auxiliary nurses. Healthcare workers and nursing assistants provide more practical assistance, such as assistance with personal and instrumental activities of daily living [14].

In exploring healthcare in the 21st century, the nature of nursing and its practical applications are of particular interest because nursing practices involve technical knowledge, rational procedures, and diverse skills [1]. A nurse’s competence includes practical understanding and personal involvement and these aspects play a vital role in the implementation of nursing practices [15, 16]. It is therefore consequential to explore nursing practices in their contexts to understand how nurses navigate their work and how organisations develop guidelines.

Existing research on nursing practices and their contexts have mainly involved inpatient care [17–19]; however, studies regarding home care practices have shown that nurses’ work is complex and extensive [20] and that nurses have a significant role in care coordination [1, 21, 22]. Nurses in home care may face challenges that are different from the hospital setting [23], navigating in working conditions adapted to a home more than a workplace, experiencing complexity and need for various expertise, being expected to work in unpredictable conditions, under time pressures, where the nurses continuously try to distribute their time fairly [1, 24]. More research is needed on nursing practices and the role of nurses in home care services, including nurses’ perceptions of their roles, what determines their practice and priorities, and how their work is organised [25–27]. Nursing plays a crucial role in home care as it interacts with several other professional groups [26]. It is practised at the interface of clinical patient care, organisational structures, and management requirements, in collaboration with other stakeholders and traditional communities of practice [17, 25, 26, 28, 29]. Home nursing may to a greater extent than in hospitals experience workloads coming from collaborating with other stakeholders in health and the need to deal with strict time resources for each patient and to navigate ambiguous goals [24, 30]. In Norway, health authorities have attempted to provide guidelines for home nursing in connection with national guidelines on comprehensive care pathways for different healthcare professions, including the care of chronically ill patients and those with complex needs [31]; they have also suggested the piloting of interdisciplinary care teams to achieve these goals [32].

Greater clarification of nurses’ personal experiences can provide important insight and encourage discussion of what nursing should entail, both in nursing education and in primary healthcare [26, 33–35]. Detailed descriptive exploratory studies of home nursing can therefore, enhance knowledge of important aspects of nursing practices. In Norway, home nursing involves many complex tasks, leading to a high workload and time pressures [36]. Home care may suffer from a lack of specialised nurses, insufficient staff, and a fast-paced work environment with a constant expansion of duties that are not necessarily followed up with relevant training courses and sufficient resources [37–40].

Regarding nurses’ professional home care practices, they can be complex due to key organisational requirements, varying goals, and differing care requirements [41]. A complex relationship can exist between theory and practice, especially in the caring professions, where one needs the ability to combine knowledge from different fields, communicate effectively and exercise discretion regarding legislation, ethics, and health policies [42, 43]. Existing studies indicate particular challenges when nursing is offered in a home care setting [44]. The developed models for complexity in nursing, such as the Chronic Care Model [45], are mostly designed for hospital care rather than home care [46]; there are also complexities involved in knowledge transfer to partners outside the profession [47, 48]. In-home care, nurses are required to utilise a multidimensional, interdisciplinary, and holistic approach. They must address factors related to patients, other health professionals, and the healthcare system such as the quality of care delivery, organisational factors, and so on [44]. The health and social care sector includes human service organisations that must deal with problems that are unexpected, unpredictable, and complex to solve, referred to as ‘wicked problems’ [29, 49]. The opposite is termed ‘tame problems’, which can be clearly defined and have obvious causes. Further, organisations offer criteria for the best solutions that can typically be used for similar problems [29]. Nurses’ hospital practices typically include many ‘invisible tasks’, which are not stated but are nevertheless important for patient safety and continuity [17–19].

Studies show that a healthcare professional’s practice tends to be based on informal ‘mindlines’ rather than solely relying on formal guidelines [50]. ‘Mindlines’ refer to a type of tacit guidelines from a socially constructed reality among nurses that include hands-on experiences from knowledge gained in a particular context. Therefore, home nursing represents a crucial context for research to achieve a deeper understanding of the nursing knowledge required for home care. The corresponding research questions are as follows: What dominates nurses’ experiences and mindsets in the context of home care nursing, and how do they solve their tasks?

## Methods

### Design

This study used a qualitative design with a phenomenological descriptive approach [51, 52]. Transcribed interview data were analysed according to stepwise induction analysis. Strict systematic empirical coding was performed to avoid preunderstandings and to seek new insight [51, 53, 54]. Through an inductive principle, we began with the transcription data and coding, moving towards concepts or theories via incremental deductive feedback loops.

The design was originally planned to involve two rounds of participant observations by nurses. However, the COVID-19 pandemic prevented this. The first author collected the data and performed the initial empirical coding. The first and second authors then conducted the analysis. All authors discussed and agreed upon the concepts. This study complies with the Consolidated Criteria for Reporting Qualitative Research (COREQ) [55].

### Participants and sampling method

The data were collected from interviews with 15 home care nurses in four Norwegian local authorities (two rural, one large urban, and one small urban). Only registered nurses were recruited as they play a leading role in the care within this context and are responsible for the coherence and coordination of daily tasks.

The inclusion criterion was at least one year of nurse employment as a home care nurse. Recruitment took place through local health management, and then via leaders of different units. Nurses who wished to participate contacted one of the researchers directly; in some cases, contact details were provided by the unit leader after prior agreement with the interested nurse. All nurses volunteering to participate were included and there were no withdrawals during the research process. All data were treated confidentially and only the research team could access them. The participant characteristics are presented in Table 1.

**Table 1:** Participant characteristics Total no. of participants: 15

	Type of local authority	Gender	Age (years)	Years practising as a Registered Nurse (RN) in home care	Nursing education profile
Nurse 1	Large urban	Female	20 − 30	4	Bachelor’s degree
Nurse 2	Large urban	Male	20 − 30	4	Bachelor’s degree
Nurse 3	Large urban	Female	20 − 30	1	Bachelor’s degree
Nurse 4	Large urban	Female	30 − 40	9	Bachelor’s degree
Nurse 5	Rural	Female	30 − 40	12	Bachelor’s degree + Psychiatric specialisation
Nurse 6	Rural	Female	60 − 70	30	Bachelor’s degree + Cancer care specialisation
Nurse 7	Rural	Male	50 − 60	10	Bachelor’s degree + Geriatric specialisation
Nurse 8	Small urban	Female	50 − 60	8	Bachelor’s degree
Nurse 9	Small urban	Female	40 − 50	16	Bachelor’s degree
Nurse 10	Small urban	Female	50 − 60	22	Bachelor’s degree
Nurse 11	Small urban	Female	60 − 70	36	Bachelor’s degree + Cancer care specialisation + Education in counselling
Nurse 12	Small urban	Female	50 − 60	26	Bachelor’s degree + Education in counselling
Nurse 13	Small urban	Female	30 − 40	5	Bachelor’s degree
Nurse 14	Rural	Female	40 − 50	20	Bachelor’s degree
Nurse 15	Rural	Female	50 − 60	14	Bachelor’s degree

### Data collection

In-depth interviews [56] were conducted between April and November 2020. In addition to interview transcripts [56], field notes were made during and between the interviews [57, 58].

All interviews were digitally recorded and transcribed verbatim. We conducted five interviews online due to COVID-19, while the others were conducted in private rooms at the participant’s place of work. The interviews lasted from 58 to 100 min (an average of 80 min). The transcribed text amounted to 371 pages. We wrote field notes and reflection notes immediately after the interviews to support the analysis.

We used a semi-structured, modifiable interview guide. Examples of questions included in the guide are as follows: ‘What is important to you as a nurse? What is important to you in your work as a nurse in home-based care? What do your work and responsibilities entail on a typical day? What do you perceive as your responsibility and role as a nurse?’. The interview guide suggested topics, however, each interviews was conducted as a normal conversation and topics in the interview guide were added if such topic became part of the conversation. This was in line with our qualitative methodology [56].

### Data analysis

We analysed the data using stepwise induction approch [51, 53]. The first step was to generate empirical data while the second was to transcribe the data. NVivo was used to facilitate this analysis. We then transferred the data to a Microsoft Word document. In this method, the analytical categories were not stipulated in advance but through a five-step process and a strict analysis from the empirical data to codes [54]. In step three, we started the empirical coding, asking the question: ‘What is the informant actually saying?’. We analysed the data by closely examining the selected statements line-by-line. Steps three and four involved a process in which we carefully analysed transcribed data to identify important sentences that answered the research question. Using a deductive code test, we checked whether the codes were generated only from the data. After this, 223 codes remained. In step four, we sorted all the codes into five thematic code groups. Table 2 shows important sentences, phrases, and essential codes. During and between the interviews and early in the analysis process, we observed the data elements leading to ideas for the analysis. We recorded these as reflection notes. These were based on spontaneous observations in the field before, during, and after the interviews [54] and were included as reference points to develop conceptual directions in the analysis [54]. In step five, we assessed the analysis concerning previous research, and concept from relevant literature. The code groups were interpreted and related to relevant concepts. We then asked the question: ‘What is this about?’. Our analysis resulted in three main concepts and patterns, which were then identified as the results of our study [54].

Tjora’s stepwise inductive method was used to ensure reliability through the creation of clear requirements for the data generation and criteria for developing codes during the analysis. The analytical process is explained in Table 2. First, the coding was kept close to the empirical core concepts and utterances, gradually becoming more abductive when analysed according to the relevant theory [54]. The analysis included a concept test [54, 59] comprising an analytical assessment of whether the concepts could ensure an ‘enduring grab’. This implies a concept that can withstand time and have a generic value while being communicable in a manner that renders it relevant and independent of the study. Reliability was sought through transparency and reflection in the research team involved during the different phases of the study [54]. The research team comprised nurses, sociologists, and both senior and junior researchers. They discussed and reflected on relevant labelling and theories at all analytical stages. In the case of disagreement, the authors discussed until an agreement was reached. The first author, a nurse, conducted the interviews and the initial coding; she had prior experience as a home nurse and spent time with the interviewees before starting the interview to achieve an effective empirical context. The goal was to elicit the informants’ experiences and become more familiar with the nursing context before the interviews’ commencement (before COVID-19 restrictions). Although participant observation was cancelled due to the restrictions of the pandemic, one strength of the study was that the researcher was able to immerse herself in the context and have close contact with the informants and their practices, views, and experiences.

**Table 2:** Relation between main categories, code grouping, and empirical codes, examples

Main category	Code grouping	Empirical codes
To be vigilant	1. All the work we do/ keeping track/ no written instructions	*There’s so much to think about, keep track of, and organise that other people don’t understand.* *We have to go through the discharge summaries to see if there’s anything unusual.* *We always have to look through the discharge summary and check and compare.* *It’s hard to describe the roundabout way you have to go from when a problem appears until it’s solved.*
	2. Working with medicine	*Working with medicines means lots of roundabout steps.*
	3. Nurse in charge/ home care management	*Being the nurse in charge is a headache, with lots of phone calls and lots to organise.*
	4. Organisation	*There’s so much bureaucracy with all the things on those lists.*
	5. Competence/ profession	*Now we have a lot more hospital work than before, I hardly know what it’s all called and there’s lots I didn’t learn in college.*
To be an all- rounder	1. All the work we do/ keeping track/ no written instructions	*It’s not written down anywhere how we can make the work go smoothly.*
	2. Working with medicine	*The patient won’t get his medicine if we don’t borrow some. So that’s what we have to do in a situation like that, we improvise…*
	3. Nurse in charge/ home care management	*If I’m the nurse in charge, I get loads of phone calls. All of them, and I have to write reports and so on.*
	4. Organisation	*There’s no money for me to have office days, so I take on responsibilities off the cuff when there’s time.* *They expected us to know how to do everything, take shifts at the nursing home, or be moved somewhere with a need, whatever it was.*
	5. Competence/ profession	*We do much more for patients in their homes than we used to, like home death, and pain pumps, and we now have both young and old patients.*
To act with independence	1. All the work we do/ keeping track/ no written instructions	*We have to do what the patient needs.*
	2. Working with medicine	*Luckily, we can borrow medicines at the nursing home even though that’s not allowed, but that’s how patients get their medicine.* *The patient won’t get his medicine if we don’t borrow some. So that’s what we have to do in a situation like that, we improvise…*
	3. Nurse in charge/ home care management	*It’s important to be responsible as a professional, and my role is to make it clear when enough is enough.*
	4. Organisation	*Our work varies a lot over time.* *There’s no money for me to have office days, so I take on responsibilities off the cuff when there’s time.*
	5. Competence/ profession	*We do much more for patients in their homes than we used to, like home death, and pain pumps and we now have both young and old patients.*

## Results

The study participants had 1 to 34 years of experience in home-based nursing. Our findings described the context of nursing in home care, what characterised the nurses’ work, how they performed their practice, and how they experience their practice within a climate of coordination.

We found three main categories as a result of the analysis: ‘To be vigilant: We have to keep checking’, ‘To be an all-rounder: We have to deal with everything’, and ‘To act with independence: Nursing in the network of practitioners’. The first two categories addressed the complexity of practising nursing in home care while the third category addressed the aspects of professional independence.

### To be vigilant: ‘We have to keep checking’

The nurses discussed their tasks, problems, and assessments throughout their workdays. These tasks are not usually written down; they arise and must be solved daily. One example is that their phones do not stop ringing and that there is always something to monitor. Many of the tasks came from the nurses’ need to be on top of things, which could represent insight referring to both tacit and implicit knowledge. Here, this knowledge was embedded in the nurses’ work processes wherein they learned through accomplishing work tasks. They described taking notes, remembering things, and ensuring that all was well in the patient’s home. This is linked to telephone calls and enquiries but also to what the nurses observe through their clinical perspectives, where they control if medicine prescriptions are specified and complete, whether information is given to the attending physician or healthcare provider, report their observations, and conduct follow-ups.

Another key area of focus for home nurses is the comparison of various documents. They compare discharge documents and medication lists with their notes, checking for changes and consistency. They may notice something unusual; often, there are inconsistencies among medication lists.

*We always have to go through the discharge summaries to check, compare, and see if there is anything unusual (Mary)*

If they find inconsistencies, these must be corrected by calling the doctor or hospital. Such errors lead to considerable additional work. In addition, medication dispensers may need to be checked and changed. Thus, the nurses’ checks prevent errors from occurring.

The nurses stated that much of their work was not visible to anyone. They felt it was difficult to explain this work to others. This is because of all the roundabout steps that are taken while solving an issue, such as receiving a phone call or observing or noticing a problem. Although the nurses found it difficult to describe the various steps they take, they believed that if the work was not completed, it would be evident.

*The result of the work is visible, but not all the roundabout steps it takes to solve problems (Mary)*

This kind of work is not reported anywhere and does not follow formal procedures. Furthermore, it may not be possible to describe all the steps, as they are often based on telephone calls, observations, or various enquiries that indicate tasks that are to be solved and completed immediately. Often, the nurse could not have predicted the issues. The nurses felt that it was crucial to do what was required in various situations. They recorded the information painstakingly to eliminate any possibility of error.

*I meet a lot of resistance, and it is a problem when my assessments suggest something’s wrong. Then, I have to fight a bit. To see whether the emergency department will do anything, for example. I call a doctor and send messages. No reply… they do not call back. We make assessments and try to argue the patient’s case, but they often do not listen. This can have serious consequences (Ashley)*

Another area where attention is paramount is medication. Every home care nurse has daily work involving patient medications. However, there is another nurse on each shift in charge of medication, who does not visit patients but works in the medication room. There are many telephone calls and messages connected to medication work and these must be answered and followed up.

The nurses stated that a patient’s doctor is not always updated on changes in medication, such as when the patient was in the emergency department or the hospital. Transitions between different healthcare facilities require extra vigilance from home care nurses, as the patient’s doctor may write and renew prescriptions in follow-up care. Nurses spend considerable time putting medicines into dispensers and checking filled dispensers daily. Multi-doses are checked against medication lists to ensure they are correct and include any changes; here, nurses know from experience that medication lists are not always consistent. This is an area that requires particular attention. If they discover a change or error in, for example, a multi-dose, they may have to remove one medicine from each pouch and replace it with one they have to remove from a filled dispenser.

*Yes, there’s a lot of work with the doctors, back and forth, you know. It’s quite a lot, we have to make sure that what they write is true. If what the patient says is true, then we do not get any message from the doctor... but that’s typical, going back and forth with these doctors. Back and forth (Olivia)*

The nurses learn to be watchful when filling dispensers and checking multi-doses, as it requires much of their time if they discover errors or inconsistencies. Calling or sending messages also takes time.

*There is a lot of roundabout work with medicines, and there are disruptions, so you can easily lose your concentration. It is a tough shift to be in the medication room, and the phones ring non-stop (Zara)*

Thus, although the work is planned for each shift, the nurses stated that many other tasks arise and must be addressed immediately.

### To be an all-rounder: ‘We have to deal with everything’

A key aspect of nursing in home care is the tasks based on decisions made in healthcare services. Typical tasks include assistance with personal hygiene, morning care, showering, measuring blood sugar, various blood tests, wound care, softening the patient’s feet, putting on elastic stockings, administering insulin, catheterisation, giving eye drops, and bringing meals. The equipment they typically bring on patient visits is an iPad (for accessing and recording information), a mobile phone, shoe covers, sanitiser, rubbish bags, and dose dispensers, alongside other equipment relevant to each patient.

The nurses described performing a much greater variety of care tasks in home nursing than they did years ago. Examples include intravenous treatment, pain pumps, terminal care, and home deaths. In the past, most patients were old, whereas today, there is a much greater variety in age and medical conditions. Thus, there have been major changes in the nurses’ work linked to various health reforms in recent years. The most important change was more advanced home treatment and other work that was previously considered ‘hospital work’.

*Now, we have a lot more hospital work than before. I hardly know what it’s all called and there’s lots I did not learn in college. (Emily)*

Many patients have complex conditions, requiring a variety of treatments and care. The nurses are challenged by new and more advanced types of home nursing, such as home palliative care, intravenous treatment, and using unfamiliar equipment. Despite the increase in specialised tasks, nurses do much of the same work as other health workers and there are often no separate lists for nurses. Typically, everyone at work may be allotted anywhere according to the list. The exception is that for each shift, the nurse in charge is responsible for significant administrative work, while there is also a dedicated medication nurse. Despite this, the other nurses also described considerable administrative work linked to patients, which was not performed in the patients’ homes but was nevertheless crucial, and tasks related to patient care.

The nurse in charge must have a good overview of the work during the shift and use a diary in which all additional tasks to be performed are written down by the appropriate date. Some of these notes are for the nurse in charge, the nurse in charge of medication, the other nurse staff, or the nurses with other administrative tasks, while others are distributed to the nurses’ visiting patients. Some use digital notebooks with pages for each patient for these additional tasks.

*The notebook can be so full of messages that we need to follow up on top of everything else. (Megan)*

*We are completely dependent on the nurse in charge of the office during the day. It’s unbelievable the amount of detail we have to focus on. (Anna)*

The nurses explained that if they are in charge, it is preferable to have a late shift the day before to make it easier to prepare for the next day. The other nurses accept that the nurse in charge delegates responsibility. The nurse in charge is frequently overworked and occasionally calls a colleague from home after her shift to ensure that nothing has been overlooked. They also sometimes must work overtime.

*There are plenty of grateful patients. I feel I am saving lives and making a difference. So, I enjoy it in spite of everything. However, I need to be very adaptable; different patients need very different care (Ashley)*

All nurses are required to be placed on multiple worklists and be familiar with all patients. As a result, they are capable of being assigned and working on any worklist. Exceptions are nurses in charge of medication and nurses with particular administrative tasks. Tasks that specifically require a nurse are often removed from the lists and passed on to the nurse in charge, or a nurse may be given extra tasks on her list. During periods with many nursing tasks, separate lists are drawn up for the nurses and, in some cases, the nurses had their own lists depending on the number of specialized tasks. The aim is for each nurse to have a certain continuity in patient care, but there is not necessarily a fixed system for this. Being familiar with many of the patients was described as an advantage in some ways because the nurses can help each other more easily. For example, they know which patients expect the nurse to arrive on time and which are flexible to any necessary change in the schedule. Another instance where ‘anyone can work on any worklist’ can be advantageous is when home care nurses gain access to all patient alarms on the daily, and are able to respond to them as necessary. Because they are familiar with the patients on their colleagues’ lists, the nurse in charge or others on duty know who can most seamlessly assist the patient or a colleague. Accordingly, they may distribute responses to alarms in a flexible way and help each other.

Disadvantages of the system of ‘anyone can work on any worklist’ are that some nurses feel insecure about not knowing where they need to report each day and find it unsatisfactory to have many tasks where they cannot use their expertise or follow up on patients beyond one shift. This affects continuity. However, various systems are used to enhance continuity, such as the diaries mentioned above, various folders, and additional lists, and as nurse Zarah exclaimed: ‘*There’s so much bureaucracy with all the things on those lists’*.

The idea that everyone should be able to work on any worklist can also mean that nurses clean floors and bring meals to patients. They do not necessarily have a negative view of this, although they may feel that their skills are not being used effectively and that resources could be utilised better.

Home care nurses perform various tasks that are not directly part of patient care. Many of these tasks are known at the start of the shift but are not included in the instructions from the healthcare service. They may come from the diary, medication folder, or any of the various lists used. Work may also arise unexpectedly during a shift. Notably, it is not always clear who is in charge of this work. In this study, a nurse discovers a deceased patient; if the death is unexpected, not only a doctor but also the police are required to confirm the death.

*Home nursing includes things you cannot prepare for, like finding someone dead. And that’s one thing, and then you think someone’s going to come and help you, but the doctor on emergency duty leaves as fast as he arrives, so then I am sitting there alone waiting for the police with a dead body I must not touch. Then, the police arrived two hours later. You might think the police would take responsibility, but they do not. I am the person left in charge, and it’s quite tough. It was not exactly a pretty sight that met my eyes; there was a lot of blood after what we think was a fall. So, I just had to take over and start calling and coordinating. Then, the police have to interrogate me with a million questions. Then, I have to contact the undertakers when the police have finished taking pictures, etc. After that, we are out of the case, and I was never told the cause of death or anything. It was unclear where the blood came from, so maybe the patient had an autopsy, but we knew nothing about that. By then, we were out of the picture (Ashley)*

Other work may include sick patients, various enquiries from specialists and general practitioners, discharges and admissions to the hospital, rehabilitation and respite, planned treatment by a specialist or therapist, patient alarms, enquiries from relatives, meetings, blood tests or injections, and other tasks that may arise. The nurses also explained that there was often extra work because they knew the patients and could anticipate problems, thereby acting to prevent them from occurring.

*I’m often in a hurry. Late for lunch and soon back again. Overtime is typical for nurses. I hardly ever see other groups doing overtime. (Ashley)*

*My family is used to me coming home late from work. (Zara)*

Weekend work usually involves discharging patients from the hospital to home just before the weekend; this often keeps nurses busy on Friday afternoons. When they start work on weekends, they know that they may have to improvise and take things as they come. They will need to check that the medicine list is correct, read the discharge summary or call the hospital if it is missing, obtain medication and equipment, handle new procedures, and assess what immediate assistance is necessary before the health service decides on healthcare for the patient. The nurses described their practice in-home nursing as varied and interesting, and they felt that they were performing nursing practice during evolving times.

### To act with independence: ‘nursing in the network of practitioners’

In home care, each nurse receives a patient list that stipulates the order in which patients are to be visited and the amount of time to be spent with each patient. However, the nurses find that in addition to the time stipulated on the list, they must spend different amounts of time with each patient to fulfil their needs.

*I do what needs doing and take the time I need. (Emily)*

*We must do what is needed for each case. (Lilly)*

The nurses do not always follow the allotted time and sometimes change the order of patients if they find it appropriate. This was based on the experience and knowledge of the patients. If they arrive late to see one patient because they have spent more time with another patient or have changed the order, they explain this to the patient. They often call the patient if they are likely to arrive late. For some patients, they avoid arriving late. This may be because care must be provided at a specific time or because certain patients are particular about their allotted time periods. Since they know which ones are firm, they must decide whether they will risk getting ‘told off’ if they arrive late. All these factors are relevant to their decisions to change the order and time spent with patients. Although the care provided is based on specific instructions from the healthcare service, it varies because different nurses observe and assess differently. Nurses also stated that they must assess patients in their homes differently than they do in hospitals. Some only provide the care stipulated in the instructions received, while others perform extra tasks out of kindness, based on a desire to do good for the patient: *“It is done out of charity… we understand when something is good for the patient” (Lilly).*

Simultaneously, as nurses feel free to make independent assessments and set priorities, there is a change in attitude toward the content of home nursing. Previously, it was closer to total care, meaning that patients received whatever kind of help they needed. In recent years, however, healthcare services have made decisions on the purpose and type of care that each patient should receive. For new patients, care is provided immediately before any decision is reached. The number of patients has increased considerably in recent years, patients have received more support for self-help, and more young patients require advanced nursing and equipment. This development involves more administrative work, but the fact that nurses need time for this work in addition to all the other work on their lists has not been acknowledged. The nurses collaborate well and call each other when they have spare time to offer help, and as nurse Cathy says: ‘*We have to help each other out*’. Quality assurance is also important for nurses. When they are unsure about some aspect of their work, they contact each other over the phone or FaceTime and learn new procedures in pairs.

Independent assessments are also linked to professional responsibility and the clinical perspective. Observation was explained as a key method in nursing care and much is revealed solely by the nurse looking at and touching a patient as she carries out the work on her list. When nurses discover challenges or changes in the patient, they know that they need to assist because there may be no one else to do it. Sometimes this work takes a long time, but their attitude is to do it expediently.

The nurses take responsibility for providing care to the patients on their list and avoid burdening the nurse on the next shift.

If they know the patient, they often have extra work because the patient more readily asks for help. One example of this is helping to prepare for admission to an inpatient facility when there are no relatives who can assist. Another example is taking blood samples to the laboratory because the results arrive faster than using regular transport. A third example is taking a urine sample because there may be early signs of infection in this particular patient. This enables the patient to start treatment sooner. When the nurse contacts a doctor, the urine sample has often already been taken. The nurse knows from experience that the patient will need treatment for infection; by starting early, she lightens the burden for the patient and family. A further example of using independent judgment is when the nurse improvises and obtains the right medication for a patient who may be sent home on a Friday afternoon from the hospital without prescriptions. The nurse may use medicine from a nursing home until the patient has received their medication. Borrowing medicines is not allowed, but in this way, the patient has their medicine on time, and the nurse has done what is best for the patient.

Observations lead to new tasks, and all these additional steps imply giving the best care to the patient. The nurses feel that they are the patient’s spokesperson and an important intermediary. In doing the best for patients, the nurses cooperate well, act as a team, and help each other. This represents a community of practice where the nurses help others, ask for and give each other advice, perform tasks, and give support to provide better care for the patients. This is also linked to their feeling of duty and professional responsibility. Sometimes the nurse’s role is to make it clear when, for example, a patient needs a higher level of care or more assistance than they can provide in their home.

## Discussion

This study explores nurse practice, what dominates nurses’ experience and mindsets, and how they solve tasks in the context of home care nursing. The findings show that nursing practice in this setting can be described in the categories of ‘to be vigilant’, ‘to be an all-rounder’, and ‘to act with professional independence’ in the network of other healthcare professionals.

The findings show that nurses continuously use their judgment in their work when there is a lack of guidelines or clear procedures to follow. As underlined by the findings and in other studies, practising nursing in a home care setting is complex. It includes not only various types of care and patients, but also multiple formal or informal stakeholders that are present and interacting with each other and with the healthcare system, and the complexity involved in situations that are not consistent or alike [44]. Existing guidelines for long-term care patients, such as the Chronic Care Model [45] or COMID [60] may not consider the full complexity of practising nursing in home care settings. Even if guidelines only represent a checklist for classifying the presence or absence of patient characteristics, various needs or care plans, recommendations, and so on, they do not fully provide the expertise needed by nurses to address complex situations. Instead, as found in this study, nurses evolve their specific way of thinking about doing the best for their patients, solving all nursing tasks under time restrictions, and finding a way to meet the complexities of home-based care.

Nursing practices involve theoretical and practical knowledge as well as the personal and professional commitment of individual nurses [1, 15]. This study’s findings show the importance of personal experience, competence in general nursing, and sensitivity toward doing nursing in a home care setting. This experience-based competence enables nurses to assess what a situation requires and act accordingly, based on both professional and conditional practical experience [1, 61]. Gabbay and le May [50], call these developing ‘clinical mindlines’. These mindlines are based on clinicians’ synthesis of knowledge from various sources to provide personalised healthcare [50, 62]. They are drawn from many sources, including the clinicians’ experience, education, advice from others, patients’ views, professional sources, colleagues, infrastructure, and opinion leaders. In this study, we find this concept relevant to the way nurses practice in a home care setting. We also find that the use of tacit knowledge [63, 64] is an element of developing mindlines [62]. Examples of actions based on tacit knowledge include that of the nurse collecting a urine sample before the guidelines even deem it necessary, starting treatment earlier, or not giving prescribed medicine until new research has been done and the doctor is consulted again. This shows how nurses in home care consider the context, alongside using their expertise learned through education or working at hospitals.

### Nurses’ vigilance and keeping on top of things

This study’s findings show that the framework of clinical mindlines evolves in the way nurses perform their patient care, including work that is unfamiliar, complex, or requires advanced procedures and equipment. In such situations, the nurses develop trajectory awareness [15] which refers to the nurses’ awareness of trajectories of care and the work of maintaining oversight. This type of mindline evolves through the experiences of patients’ needs and changes in those needs. They solve tasks based on their experience in providing home nursing to various patient groups with different care needs. A type of mindline is developed in which nurses in this practice decrease their dependence on specialists to perform their work. The knowledge they acquire from this work makes them all-rounders that are prepared for most eventualities. They need to handle various tasks and organise and coordinate patient care, making them robust and capable of adapting to new tasks and situations; this is in line with other research in home care [1, 61, 65]. This encompasses what Allen [66] calls trajectory articulation, which refers to practises that ensure all of the elements necessary to meet patient needs and where the work process combines the actions, knowledge, and resources needed to provide high-quality home care.

Additionally, the findings show that home nursing is not only complex but also requires professional independence in addition to professional judgment in complex situations. When the nurses received worklists that stipulated the order and times of patient visits, they made their own assessments of what they think is best and most appropriate for the nursing tasks involved. In this way, they resolved any contradictions between the work required and the time allotted to it, performing what they considered to be holistic, individual, and caring work. There have been studies claiming that, due to the complexity of care and time constraints, nurses may compromise their quality of care [24]. In this study, however, we found that nurses make individual or collective mindlines toward care quality. Practising nursing in a home care setting implies an endless journey of fulfilling tasks and meeting requirements for care. This journey is both unforeseen and demanding, requiring professional judgements [67, 68].

This study shows that it was necessary to interact with various doctors and healthcare personnel when a patient was discharged. It was particularly important to pay attention when coordinating different treatments and medication lists. Experience showed that nurses needed to be alert to avoid extra work or negative consequences for patients. The nurses in this study untied various ‘nodes’, such as checking dose dispensers and multi-doses to prevent incorrect medication after a visit to a doctor or hospital. This requires experience-based action that nurses develop through their understanding of the context and potentially risky situations. Nurses can work in teams with other healthcare professionals, alone, or in consultation with patients. The findings show the importance of home care nurses understanding the complexity of the nursing context, namely, balancing different resources and needs and being able to prioritise and manage home care as an ‘emergent organization’ [18, 66]. Nevertheless, even if studies show that clients have little influence on which tasks are carried out and how much time is allotted to them when it comes to the prioritizing of care [24, 68], and that there exist tight schedules that seldom allow the time needed for doing holistic care [24], the nurses in home care settings use their expertise in a context-sensitive way. The nurses in this study demonstrate both contextual awareness and what Gabbay & le May [62] call ‘contextual adroitness’, despite limited time and the complexity of practising home care nursing.

### Nurses as all-rounders

The ability to manage emergent organisations, like home care, can also shed light on another main concept in this study, namely nurses as all-rounders. Home nursing is a complex service [1, 44, 60, 69] that includes both emergencies and chronically ill patients with complex diseases and care needs. Further, this can be linked to the system of ‘everyone works on any worklist’. A nurse’s work may range from dealing with home death and complex medical treatment and equipment to delivering meals and cleaning floors. This requires considerable flexibility. Much of the work is described as ‘wicked problems’ [49, 70], and the nurses do various tasks from administering care, arranging the patient’s clothes, or taking the patient to the doctor if that is what the nurse considers is required for holistic care. Thus, home care nurses create their own preparedness plans, acting as coordinators to ensure that they do not burden others with the responsibility. An example in this study is a nurse who stays with a dead patient before the undertaker takes over the responsibility.

### The nurses’ professional independence

Nursing is an adaptive complex system [71], particularly in the home care setting [44]. Theories about nursing may lack focus on integrated care, and development about mindlines in the home care context is poorly developed both in nursing theories and frameworks for practising nursing [72]. The findings of home care nurses’ professional independence are relevant in understanding the context of home care. In this study, we found that nurses struggle to maintain professional autonomy in a care setting that is complex and less regulated by clinical guidelines. The nurses must use their own professional judgement and evolve their clinical mindlines for providing quality care. They do what they believe is right based on their experience, sensitivity to the complexities involved, and knowledge about the patient. Other factors include patient care needs; clinical guidelines about treatments and medication; and information about the home setting, culture, religion, and so on. Such findings coincide with the fact that individual nurses must prioritise their time during their workdays [24], playing a key role in the coordination of care [25].

In this study, we found that nurses share values and priorities with nurse colleagues. They did not only carry out job tasks according to formal decision-making, regulations, or instructions from the healthcare service. This may seem surprising considering home care is a regulated practice with specified procedures for providing care [27, 73].

The clinical mindlines used by nurses practising in home care settings are based on working and practising in a certain context. Nurses in home care settings identify with a community of practices wherein they work in a specific way. This working practice comes from a community that, over time, collaborates during breaks, in the car, in various meetings, and when writing reports. They address issues concerning patients or patient groups, diagnoses, or medication management in a specific way, learned by the community of nurses practising in home care settings. The nurses help each other with demanding tasks and work collectively, found to be essential in home-based care [1]. A professional network is formed, where home care nurses work hard to introduce, transform, and integrate knowledge based on their experience in this practice [74–76]. Such informal interactions create a community among nurses who share the same nursing context. In this way, both collective and individual mindlines could be said to develop, enabling nurses to meet complex and often conflicting demands as best they can [50, 62]. These mindlines are interwoven with a type of community of practice characterised by a ‘social life of knowledge’, as described by Brown and Duguid [77]. Communities of practice are formed by various enabling and constraining factors that nurses encounter in their organisation, relationships, processes, experiences, skills, interactions, and positions [78]. This study shows that nursing practice in home care requires the use of different knowledge and insights into the complexity involved. Practice is thus linked to ‘knowledge in practice in context’ [56] where nurses base their decisions and actions on what they find works well, almost independently of the overall organisation and its healthcare decisions.

### Strengths and limitations

This study was conducted in Norway, so it may be difficult to transfer the results to other cultures and contexts with different organisations of healthcare services. The transferability of the findings could be a limitation of this study. This study provides an understanding of nursing practice in home nursing, how nurses navigate their work, and how they develop their practice and their contextual understanding.

Nevertheless, the aim of qualitative research is not to extend findings derived from selected samples to the world at large but rather to transform them and apply them to similar situations in other contexts [79].

## Conclusion

Nurses use clinical mindlines for practising nursing. Nurses’ contextual awareness and adroitness are crucial to the way they practice nursing and solve tasks. They need to manage emergent organisations and exercise professional independence and judgment when adapting their work to this context. The home nursing practice involves clinical mindlines, where knowledge in practice in context (tacit and explicit knowledge of the nurse and their colleagues) forms the basis for home nursing practice. Additionally, a community of practice evolves among nurses sharing the same experience of practising in home care.

Further studies are needed to determine more knowledge about the characteristics of clinical mindlines and for both nurses and health care workers in the context of home care. As previous research shows, we also found that home care settings are very complex. Our study shows that nurses used discretion and professional independent judgment to solve tasks and carry out their work, which requires a vigilant attitude. This study can contribute to future health policy; home care should not only be based on standardised guidelines. To provide holistic and individual care, nurses must be allowed to practice nursing with a background in local knowledge and clinical mindlines in context.

## Data Availability

The data used in this study are available from the corresponding author upon reasonable request.

## Abbreviations

COREQ: Consolidated Criteria for Reporting Qualitative Research
